# miRNAs as markers for the development of individualized training regimens: A pilot study

**DOI:** 10.14814/phy2.15217

**Published:** 2022-03-11

**Authors:** Manuel Widmann, Felipe Mattioni Maturana, Christof Burgstahler, Gunnar Erz, Philipp Schellhorn, Annunziata Fragasso, Angelika Schmitt, Andreas M. Nieß, Barbara Munz

**Affiliations:** ^1^ Department of Sports Medicine University Hospital Tübingen Tübingen Germany; ^2^ Interfaculty Research Institute for Sports and Physical Activity Eberhard Karls University of Tübingen Tübingen Germany

**Keywords:** individual training adaptation, microRNAs, physical exercise, skeletal muscle

## Abstract

Small, non‐coding RNAs (microRNAs) have been shown to regulate gene expression in response to exercise in various tissues and organs, thus possibly coordinating their adaptive response. Thus, it is likely that differential microRNA expression might be one of the factors that are responsible for different training responses of different individuals. Consequently, determining microRNA patterns might be a promising approach toward the development of individualized training strategies. However, little is known on (1) microRNA patterns and their regulation by different exercise regimens and (2) possible correlations between these patterns and individual training adaptation. Here, we present microarray data on skeletal muscle microRNA patterns in six young, female subjects before and after six weeks of either moderate‐intensity continuous or high‐intensity interval training on a bicycle ergometer. Our data show that *n* = 36 different microRNA species were regulated more than twofold in this cohort (*n* = 28 upregulated and *n* = 8 downregulated). In addition, we correlated baseline microRNA patterns with individual changes in VO_2_max and identified some specific microRNAs that might be promising candidates for further testing and evaluation in the future, which might eventually lead to the establishment of microRNA marker panels that will allow individual recommendations for specific exercise regimens.

## Graphical abstract

Using microarrays and qPCR analysis, we studied microRNA patterns in skeletal muscle in healthy subjects undergoing two different six‐week exercise programs. We identified several miRNAs that had not been implicated in exercise adaptation before. In addition, our data suggest that exercise‐induced changes in microRNA patterns are dependent on exercise type and also on individual background, a finding that suggests that microRNAs might serve as biomarkers for the development of individualized training regimens in the future.

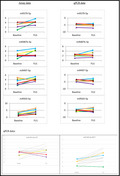

## 1 INTRODUCTION

Exercise induces a broad variety of adaptation reactions in multiple tissues and organs, such as the respiratory and cardiovascular systems, as well as in skeletal muscle tissue. These adaptation reactions are induced by exercise‐specific physiological changes, such as mechanical stretching, energy depletion, calcium oscillations accompanying muscle contraction, or systemic factors such as pro‐inflammatory cytokines or hormones. All of these factors can induce changes in gene expression patterns (for review, see Wackerhage & Woods, 2002). However, the mechanisms by which these alterations are regulated are incompletely understood. Specifically, little is known on individual‐specific factors that determine qualitative and quantitative aspects of training adaptation, i.e., that underlie the trivial observation that different individuals may react very differently to the same training regimen.

Recently, a specific group of short, non‐coding RNAs, so‐called microRNAs (miRs) is getting more and more attention. It has been known for a while that miRs can regulate concentrations of their unique spectrum of target mRNAs in a well‐controlled and very specific manner. The respective mechanisms are complex and still not completely understood, ranging from mRNA de‐stabilization, which appears to be the most important mechanism in mammalian cells (for review, see Guo et al., 2010), to modulation of transcription and translation. With regard to skeletal muscle biology, miRs have specifically been shown to play a major role in the regulation of the IGF‐1/AKT/mTOR/FoxO1 and myostatin pathways, thereby controlling the balance between myogenesis/muscle hypertrophy and muscle protein degradation (for review, see Hitachi & Tsuchida, 2014). miRs can be found within cells, but also extracellularly (e.g. in the circulation) or in exosomes (for review, see Jiménez‐Avalos et al., 2021), and recent work by Van Pelt et al. (2020) suggests that specifically extracellular miR‐203a‐3p might play a major role in the regulation of skeletal muscle growth and atrophy.

Whereas many miRs are present in a broad variety of tissues and organs, others are tissue‐ or even cell type‐specific (for review, see Gargalionis & Basdra, 2013). For skeletal and/or heart muscle‐specific miRs, the term myomiRs has been coined. In general, miRs ‐1, ‐133a, ‐206, ‐208a, and ‐499 are considered as myomiRs. Several authors also include miR‐486, which is, despite being found at high concentrations in muscle cells, not muscle‐specific (for review, see Horak et al., 2016; Luo et al., 2013; McCarthy, 2011).

While plasma‐based approaches might eventually be more suitable strategies in training practice, analysis of miRs directly from skeletal muscle tissue, i.e., from the tissue that is the major player in the context of physical activity, might lead to a deeper understanding of the molecular mechanisms by which miRs shape exercise adaptation.

To date, only a few studies on miR regulation in skeletal muscle in response to exercise interventions are available (for review, see Silva et al., 2017, 2020; Widmann et al., 2019). The results are difficult to compare, since there is a lot of heterogeneity, for example with respect to organism (human vs. animal), training modality (e.g., endurance vs. resistance exercise, moderate‐intensity continuous training [MICT] vs. high‐intensity interval training [HIIT]), or duration of the respective intervention, ranging from single bouts of “acute” exercise to several weeks of regular training. Some studies only analyzed the concentration of selected miR species, predominantly myomiRs, whereas, in others, broad‐range, unbiased approaches such as miR arrays were employed. Nevertheless, the results suggest that certain miRs might be central players in the process of exercise adaptation—and that their targets regulate a broad variety of crucial aspects of the adaptation process, such as skeletal muscle hypertrophy, angiogenesis, or mitochondrial biogenesis. Examples are miR‐1, miR‐23, miR‐26a‐5p, miRs‐29a, and ‐29b‐3p, miRs ‐133a and ‐133b, miR‐206, miR‐378, miR‐451, or miR‐696 (Aoi et al., 2010; Davidsen et al., 2011; Drummond et al., 2008; Fyfe et al., 2016; Keller et al., 2010; McCarthy & Esser, 1985; Mueller et al., 2011; Nielsen et al., 2010; Ogasawara et al., 2016; Rivas et al., 2014; Rowlands et al., 2014; Russell et al., 2013; Safdar et al., 2009). However, to determine the factors that are responsible for the regulation of miR patterns by exercise, it will be necessary to analyze, determine and compare these effects in standardized settings, such as metabolically controlled MICT and HIIT regimens.

In addition, so far, only a few of these studies have analyzed potential correlations between miR patterns and individual exercise‐associated physiological parameters: Whereas some authors have correlated patterns of circulating miRs and relative maximum oxygen uptake (VO_2_max) (Baggish et al., 2011; Bye et al., 2013; Li et al., 2018), only one study has done so for skeletal muscle miR patterns and the individual degree of training‐induced physiological changes, specifically increases in muscle mass (Davidsen et al., 2011). Still, the results of these few studies indicate that there might indeed be associations between miR patterns in skeletal muscle and training‐induced physiological changes. Furthermore, to our knowledge, there aren't any studies analyzing correlations between concentrations of specific miRs in skeletal muscle at baseline, i.e., before the start of the intervention, and final physiological outcome, specifically changes in VO_2_max, so far. Nevertheless, such correlations could be particularly interesting, since they might allow prediction of an individual's response to training in general and/or a specific training regimen, thus helping to design and plan training schedules in competitive as well as in therapeutic and rehabilitative settings.

Against this background, we were aiming at resolving the following questions: (1) Which miRs are differentially expressed in response to two metabolically controlled, standardized endurance exercise settings? and (2) Can individual miR patterns be correlated with a specific subject's degree of training adaptation?

As an initial, exploratory, hypothesis‐generating approach, we employed miR microarray analysis to study miR patterns in skeletal muscle tissue of six young, female, healthy, sedentary subjects before and after completion of two different endurance‐based exercise regimens. miR microarray analysis represents a comprehensive, unbiased approach, however, is also elaborate and expensive, thus, the number of samples that can be analyzed using this method is limited. Against this background, we selected a group of all‐female subjects which was quite homogeneous specifically with regard to age, BMI, and baseline VO_2_max. Thus, the study's goal was the generation of initial hypotheses, which should be verified and confirmed via inclusion of more data in the future.

## 2 MATERIALS AND METHODS

### 2.1 Subjects and training intervention

The sub‐project described here is part of a larger study, the so‐called iReAct (“individual response to physical activity”) project, that analyzes individual biopsychosocial aspects of exercise adaptation (study registration in the German Clinical Trials Register on June 12, 2019 [DRKS00017446]). This study meets the ethical standards as stated by Harriss et al. (2019) (Ethics approval by the ‘Ethics Committee of the Medical Faculty, University of Tübingen’ on January 22, 2018 [reference number: 882/2017BO1]), and written informed consent was obtained from all participants before inclusion. The design of the study has previously been described (Mattioni Maturana et al., 2021; Maturana et al., 2021; Thiel et al., 2019). Briefly, sedentary, healthy, young (20–40 years) participants (male and female, *n* = 42, which is a COVID 19‐associated deviation from the originally planned *n* = 60) completed two six‐week blocks of aerobic exercise training on a bicycle ergometer. Because of the under‐representation of males in the limited study cohort, for the sub‐project described here, only data from female participants were analyzed. They were randomly assigned to either starting with MICT (moderate‐intensity continuous training) or HIIT (high‐intensity interval training). Subjects completed three sessions of 60 min (MICT) or 43 min (HIIT) per week, with relative total workload calculated to be equal for both training forms. Participants doing the MICT program performed 60 min of continuous cycling at the power output corresponding to 90% of the first lactate threshold (LT1) (Binder et al., 2008; Hofmann et al., 1997; Pokan et al., 1997), whereas the HIIT group began with a warm‐up for 10 min at the power output equivalent to 70% of the maximum heart rate (HRmax), followed by four 4‐min intervals, each at the power output corresponding to 90% of HRmax. In between the intervals, subjects did a 4‐min, active recovery at 30 W (MacInnis & Gibala, 2017). MICT was based on LT1 to ensure that exercise intensity was reliably within the moderate‐intensity domain: Since we did a step incremental test rather than a ramp, analyzing through the lactate thresholds was more appropriate than via ventilatory thresholds. By contrast, HIIT was prescribed based on % HRmax and not LT2 because of the training monitoring throughout the weeks: Since sedentary participants tend to improve quickly, we needed to make sure that they were always exercising within the severe‐intensity domain. Therefore, since heart rate was monitored in every training session, we could make sure that we would increase the intensity throughout the weeks, so that they were always exercising at an intensity that would produce approximately 90% HRmax. This was consistent with our aim that participants would be exercising between 90% and 95% HRmax. That way, we made sure that exercise intensity retrieved from 90% HRmax would be greater than the intensity at LT2. If this had not been the case for specific participants, we would have prescribed intensities corresponding to 95% HRmax. In our sample, however, all participants were above LT2 at 90% HRmax, so we did not have to make this adjustment (Maturana et al., 2021). Before the intervention and after both training blocks (baseline and follow‐ups FU1 and FU2), diagnostics was performed as described (Thiel et al., 2019), including a broad variety of tests, aiming at a holistic, biopsychosocial perspective on exercise adaptation. Anthropometrical measures, which included height, weight, and bioimpedance analysis (InBody770, InBody), were taken for estimation of body composition (i.e., body fat and muscle mass) at each of these time points. To minimize the risk of acute effects of the last training session, subjects were subjected to a break of at least 48 h between their last training session and FU1/FU2 diagnostics, which also included the biopsy sample timepoints. After the first six‐week block (and FU1), subjects were switched to the respective other training regimens without a wash‐out period. Skeletal muscle biopsies from the vastus lateralis muscle were taken before the start of the intervention, after the first six weeks of training, and at the end of the intervention. Samples were immediately snap‐frozen in liquid nitrogen and stored at −80°C until further use. Furthermore, physiological markers for training adaptation, particularly maximum relative oxygen uptake (VO_2_max) were assessed. For the explorative subproject described here, we analyzed biopsies from six female participants (age 20–29 years, VO_2_max 31.7 ± 1.6 ml kg^−1^ min^−1^; for detailed subject characteristics, see Table 1) before the intervention and after the first training block by miR microarray analysis (cf. 2.3), with three subjects doing HIIT and three doing MICT. For qPCR analysis (cf. 2.3), a larger cohort of female individuals (age 27.4 ± 6.4 years, MICT: *n* = 13, HIIT: *n* = 12, VO_2_max 29.76 ± 3.27 ml kg^−1^ min^−1^) was analyzed.

**TABLE 1:** Subject characteristics

Participants	Age (years)	VO_2_max Baseline (ml kg^−1^ min^−1^)	VO_2_max FU1 (ml kg^−1^ min^−1^)	height (cm)	weight (kg)	BMI (kg m^−2^)	Training
**HIIT**							
#IR 0008 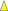	20	32.75	37.67	164.5	59.3	21.9	HIIT
#IR 0012 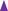	21	29.18	33.26	173.5	68.9	22.9	HIIT
#IR 0042 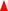	27	30.80	35.22	160.4	57.6	22.4	HIIT
Mean	22.67	30.91	35.38	166.13	61.93	22.4	
SD	3.09	1.46	1.80	5.47	4.97	0.41	
**MICT**							
#IR 0005 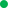	22	33.24	38.92	167.5	61.2	21.8	MICT
#IR 0010 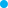	21	30.64	33.97	163	66.5	25	MICT
#IR 0030 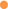	29	33.55	36.54	159	60.9	24.1	MICT
Mean	24	32.48	36.48	163.17	62.87	23.63	
SD	3.6	1.30	2.02	3.47	2.57	1.35	
**All**							
Mean	23.33	31.69	35.93	164.7	62.4	23.0	
SD	3.40	1.59	1.99	4.82	3.99	1.17	
*p*‐value		0.43	0.05	0.82	0.06	0.11	

### 2.2 VO_2_max analysis

A step‐incremental test to volitional exhaustion was performed during baseline and both follow‐ups for VO_2_max assessment as described (Mattioni Maturana et al., 2021; Maturana et al., 2021). Briefly, participants performed the protocol on a bicycle ergometer (Ergoselect 200, Ergoline GmbH) and heart rate and electrocardiogram were monitored continuously (12‐channel PC ECG, custo med GmbH). The test started with a 2‐min resting period on the bike, followed by 3‐min steps. Female participants started cycling at 25 W (male participants—not included in the present manuscript—started at 50 W) and each step was increased by 25 W. Breath‐by‐breath pulmonary gas exchange and ventilation were measured using a metabolic cart (MetaLyzer, CORTEX Biophysics). Calibration was performed before each test following the manufacturer's instructions. Breath‐by‐breath VO_2_ data were edited as follows: breaths (data points) that were two standard deviations (95% of confidence interval) away from the local mean were considered outliers and then removed (Lamarra et al., 1987). Thereafter, the data were interpolated on a second‐by‐second basis and averaged into 30‐s bins. VO_2_max was considered the highest 30‐s VO_2_ average. VO_2_max attainment was confirmed if at least one of the following criteria were met, as per the American College of Sports Medicine guidelines (Riebe et al., 2018): (1) maximal heart rate within 10 beats per minute (bpm) of the maximal predicted value (220–age); (2) a respiratory exchange ratio (RER) higher than 1.10; or (3) a maximal blood lactate concentration of 8 mmol L^−1^.

### 2.3 RNA isolation and miR analysis

To assess miR concentrations before and after the training intervention, total skeletal muscle RNA, including miR, was extracted from baseline (before training) and FU1 (after the first six weeks of training) specimens using the miRNeasy Micro Kit (Qiagen) according to the manufacturer's instructions. Quantity and purity of RNA were assessed (260/280 ratio) using a BioPhotometer (Eppendorf AG). Affymetrix miR Array 4.0 (Thermo Fisher; #902411; 2578 human mature and 2025 human pre miRs) analysis was carried out by ATLAS Biolabs, Berlin, Germany, according to the manufacturer's instructions. Data were analyzed for differential miR expression using the transcriptome analysis console (TAC), version 4.0.2.15. Based on the data format generated by this software, throughout the presentation and discussion of our data, wherever possible, we refer to individual miRs using their complete name (including “strand specification”, such as “‐5p” or “‐3p”). For qPCR analysis, 400 ng of total RNA/miR were used for reverse transcription using the miScriptII Kit (Qiagen) in combination with HiSpec buffer in a total volume of 20 µl. The cDNA was diluted and employed in qPCR analyses using the miScript SYBR Green kit from Qiagen according to the manufacturer's instructions. Primers were purchased from Qiagen (Table 2). Relative expression levels were calculated using the comparative CT (2^−ΔΔCT^) method, where expression was normalized to SNORD95 and SNORD96A.

**TABLE 2:** Primers employed in qPCR analysis

miRNA	Primer catalog Number QIAGEN
miR‐1	MS00008358
miR‐21‐5p	MS00009079
miR‐133a‐3p	MS00031423
miR‐133b	MS00031430
miR487b‐3p	MS00004298
miR503‐5p	MS00033838
miR‐497‐5p	MS00004361
miR‐379‐5p	MS00009653
SNORD95^a^	MS00033726
SNORD96A^a^	MS00033733

^a^ Served as housekeeping genes.

### 2.4 Correlation analysis

To identify possible correlations between baseline miR patterns and individual training‐induced adaptations, namely VO_2_max, we employed Spearman correlation analysis, which is less sensitive to outliers specifically in small sample sizes than Pearson correlation (Schober et al., 2018).

### 2.5 KEGG pathway analysis

KEGG (Kyoto Encyclopedia of Genes and Genomes) pathway analysis (Vlachos et al., 2015) was carried out using the DIANA‐miRPath v.3 platform.

### 2.6 Statistical analysis

qPCR data for the larger cohort (MICT: *n* = 13, HIIT: *n* = 12) were analyzed using SPSS software (Version 27; IBM). Data were considered significant with *p*‐values of less than 0.05 (*) or less than 0.01 (**). Data are presented as means ± SD. “*n*” represents the number of subjects in each group.

## 3 RESULTS

The criteria for VO_2_max attainment were met by all six participants: (1) maximum heart rate ranged from 191 to 200 bpm at baseline, and from 189 to 203 bpm at FU1 (age‐predicted maximum heart values ranged from 191 to 200 bpm); (2) the RER ranged from 1.16 to 1.33 at baseline, and from 1.23 to 1.28 at FU1; (3) maximal blood lactate concentration ranged from 6.8 to 11.7 mmol L^−1^ at baseline and from 8 to 11.7 mmol L^−1^ at FU1. VO_2_max improved from (values are mean ± standard deviation unless otherwise stated) 31.7 ± 1.6 ml kg^−1^ min^−1^ at baseline to 35.9 ± 2.0 ml kg^−1^ min^−1^ at FU1. VO_2_max values at baseline ranged from 29.2 to 33.5 ml kg^−1^ min^−1^ (mean = 31.7 ml kg^−1^ min^−1^); and from 33.3 to 38.9 ml kg^−1^ min^−1^ (mean = 35.9 ml kg^−1^ min^−1^) at FU1 (Table 1). Individual maximum values are reported in Table 3. There were no statistically significant differences between subjects of the MICT and HIIT groups with regard to baseline parameters, however, differences for VO_2_max improvement almost reached significance, with HIIT training resulting in greater improvements (Table 1; *p*‐value = 0.05489). This could be confirmed when data from all participants were analyzed (Mattioni Maturana et al., 2021; Maturana et al., 2021).

**TABLE 3:** Criteria for VO_2_max attainment for all six subjects before and after training as indicated

Id	Absolute VO_2_max (L/min)		Relative VO_2_max (mL/kg/min)		Criteria for VO_2_max attainment								
					Age‐predicted HRmax (bpm)	HRmax (bpm)		RER		Maximal blood lactate concentration (mmol/L)		Criteria met	
	Pre	FU1	Pre	FU1		Pre	FU1	Pre	FU1	Pre	FU1	Pre	FU1
IR0005	2.03	2.39	33.2	38.9	198	197	197	1.21	1.28	8.42	9.85	✓HRmax, ✓RER, ✓Lactate	✓HRmax, ✓RER, ✓Lactate
IR0008	1.95	2.28	32.8	37.6	200	200	203	1.25	1.28	8.63	7.95	✓HRmax, ✓RER, ✓Lactate	✓HRmax, ✓RER, ✓Lactate
IR0010	2.04	2.23	30.7	34.0	199	200	203	1.16	1.23	7.77	7.95	✓HRmax, ✓RER	✓HRmax, ✓RER, ✓Lactate
IR0012	2.01	2.25	29.1	33.2	199	191	189	1.30	1.26	6.78	11.7	✓HRmax, ✓RER	✓HRmax, ✓RER, ✓Lactate
IR0030	2.04	2.19	33.5	36.2	191	195	197	1.30	1.26	11.69	10.02	✓HRmax, ✓RER, ✓Lactate	✓HRmax, ✓RER, ✓Lactate
IR0042	1.77	1.94	30.8	35.0	193	198	194	1.33	1.23	11.09	9.08	✓HRmax, ✓RER, ✓Lactate	✓HRmax, ✓RER, ✓Lactate
Mean	1.97	2.21	31.7	35.8	197	197	197	1.26	1.25	9.06	9.43		
SD	0.10	0.15	1.8	2.2	4	3	5	0.06	0.02	1.92	1.43		
Median	2.02	2.24	31.8	35.6	199	198	197	1.27	1.26	8.53	9.47		
Min	1.77	1.94	29.1	33.2	191	191	189	1.16	1.23	6.78	7.95		
Max	2.04	2.39	33.5	38.9	200	200	203	1.33	1.28	11.69	11.70		

With regard to training intensities, the HIIT group showed an average of 90 ± 3% HRmax, whereas the MICT group presented at 69 ± 6% HRmax. In relation to the work performed, the HIIT group had a relative total work of 3.1 ± 0.4 kJ/kg, and the MICT group of 3.3 ± 0.7 kJ kg^−1^ (*p* = 0.27).

Subjects’ body composition did not change significantly during the intervention, with body weight ranging from 57.6 to 68.9 kg before and from 55.4 to 68.0 kg after the intervention, and total body fat ranging from 23.5% to 32.2% before and from 24.2% to 34.3% after the intervention (Table 1 and data not shown).

**FIGURE 1:**
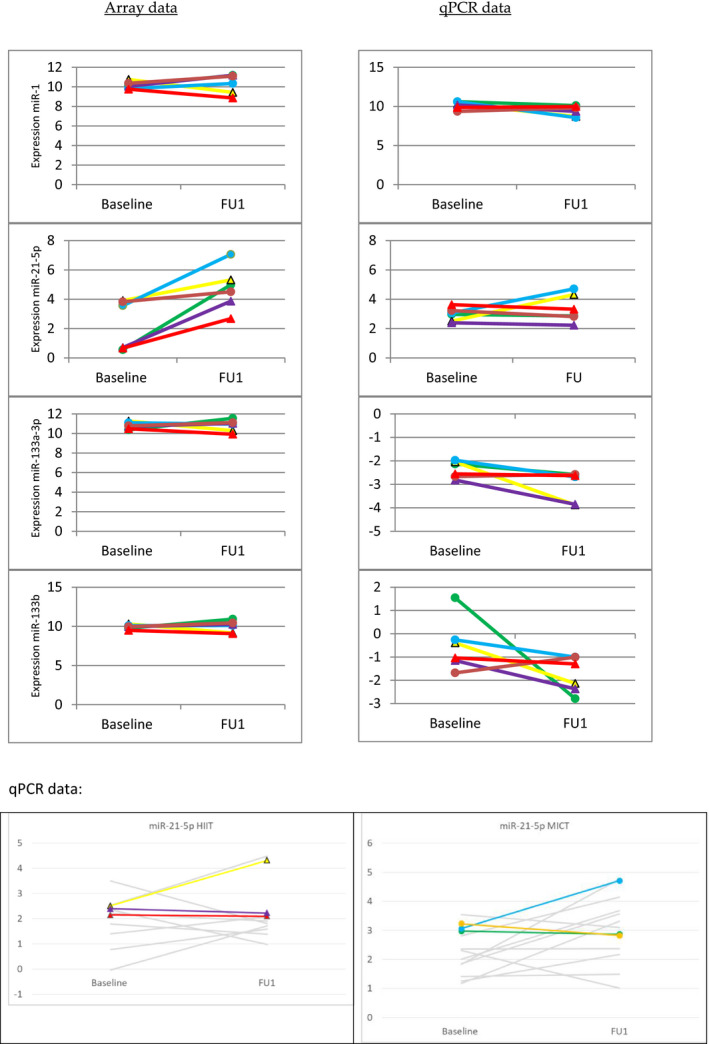
Regulation of miRs ‐1(‐3p), ‐21(‐5p), ‐133a(‐3p), and ‐133b in skeletal muscle samples of all six subjects in response to exercise. Normalized array data (log2‐transformed) are shown for all participants (left panels), right panels show corresponding qPCR data. Circles mark subjects that performed MICT, triangles subjects that performed HIIT training. Bottom panels show qPCR data for miR‐21‐5p in a larger cohort of subjects (MICT: *n* = 13, HIIT: *n* = 12). Left and right panels represent subjects performing HIIT and MICT, respectively. Grey lines represent subjects only included in the qPCR, but not in the microarray analysis. miR‐21‐5p was regulated 1.46‐fold (*p* = 0.028*), and was stronger in subjects performing MICT (1.73‐fold; *p* = 0.038*; *n* = 13) when compared to HIIT (1.19‐fold; *p* = 0.471; *n* = 12)

Based on the fact that their expression has been analyzed in several training studies and in different tissues and organs, as well as in the circulation, before (for review, see Gargalionis & Basdra, 2013; Lamarra et al., 1987; Luo et al., 2013; McCarthy, 2011), at first, miR‐1(‐3p) (muscle‐specific), miR‐21(‐5p), miR133a(‐3p) (muscle‐specific), and miR‐133b were selected for further analysis in our explorative study. As shown in Figure 1, array, as well as subsequent qPCR analysis, suggested no regulation of miR‐1 expression by training. By contrast, array analysis indicated moderate to strong induction of miR‐21 by training in all subjects. Using qPCR analysis, we could confirm and quantify this result in a larger cohort of all‐female subjects (MICT: *n* = 13, HIIT: *n* = 12). Here, overall, induction of miR‐21‐5p was 1.46‐fold (*p* = 0.028), and was stronger in subjects performing MICT (1.73‐fold; *p* = 0.038*) when compared to HIIT (1.19‐fold; *p* = 0.471) (Figure 1). By contrast, for miRs ‐133a and ‐133b, array data suggested no regulation, whereas there were obvious trends toward downregulation in the qPCR analysis (Figure 1).

**FIGURE 2:**
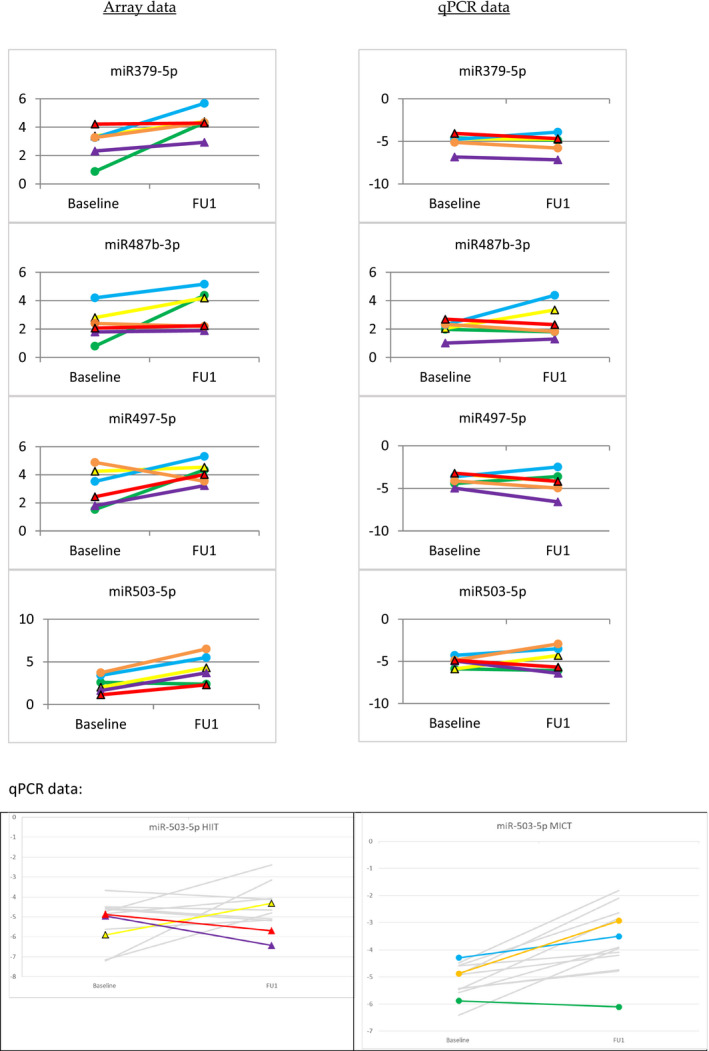
Expression patterns of miRs ‐379‐5p, ‐487b‐3p, ‐497‐5p and ‐503‐5p as assessed by miR microarray and qPCR analysis. Circles mark subjects that performed MICT, triangles subjects that performed HIIT training. Bottom panels show qPCR data for miR‐503‐5p in a larger cohort of subjects (MICT: *n* = 13, HIIT: *n* = 12). Left and right panels represent subjects performing HIIT and MICT, respectively. Grey lines represent subjects only included in the qPCR, but not in the microarray analysis. Overall induction was 2.15‐fold (*p* = 0.002**), and was stronger in subjects performing MICT (2.82‐fold; *p* = 0.004**; *n* = 13) when compared to HIIT (1.58‐fold; *p* = 0.188*; *n* = 12)

Furthermore, our array data indicated up‐ or downregulation of several other miRs, with some of them previously not having been implicated in exercise adaptation of skeletal muscle tissue. Table 4 demonstrates that *n* = 36 mature miRs, among them miR‐21‐5p, were up‐ or downregulated more than twofold on average in all subjects, whereby *n* = 28 miRs were up‐ and *n* = 8 miRs were downregulated. When comparing the effects of MICT and HIIT, we found that some of the miRs were regulated by MICT or HIIT only, whereas others were regulated by both training regimens (Table 4). qPCR analysis of four individual miR species, which were selected from the list based on literature and own, unpublished data on potential relevance in exercise and the aim of covering a broad spectrum of fold changes, ranging from 2.07 for miR‐379(‐5p) to 5.58 for miR‐21(‐5p), with qPCR data for the latter being displayed in Figure 1, indicated partially consistent results, specifically for miRs ‐487b‐3p and 503‐5p, which in case of the latter could also be reproduced in a larger cohort of subjects (MICT: *n* = 13, HIIT: *n* = 12, see above) (Figure 2). Here, overall, induction of miR‐503‐5p was 2.15‐fold (*p* = 0.002*), and again was stronger in subjects performing MICT (2.82‐fold; *p* = 0.004**) when compared to HIIT (1.58‐fold; *p* = 0.188). By contrast, upregulation of miRs 379‐5p and ‐497‐5p could not be confirmed by qPCR analysis (Figure 2).

**TABLE 4:** miRs with mean up‐ or downregulation of more than twofold by either MICT or HIIT or by both types of exercise as analyzed by microarray analysis in skeletal muscle samples of all six subjects

Transcript‐ID miRNA	Baseline Avg (log2)	FU1 Avg (log2)	Fold change	Training
hsa‐miR‐8063	2.75	1.09	−3.17	HIIT/MICT
hsa‐miR‐3188	2.96	1.47	−2.82	MICT
hsa‐miR‐619‐5p	4.23	2.86	−2.59	HIIT/MICT
hsa‐miR‐1180‐3p	3.83	2.54	−2.44	HIIT/MICT
hsa‐miR‐6790‐5p	4.46	3.22	−2.37	MICT
hsa‐miR‐6806‐3p	4.65	3.44	−2.32	MICT
hsa‐miR‐339‐3p	2.63	1.55	−2.12	MICT
hsa‐miR‐5000‐5p	1.92	0.91	−2.02	^a^
hsa‐miR‐379‐5p	3.28	4.33	2.07	HIIT/MICT
hsa‐miR‐199a‐3p	7.58	8.63	2.07	MICT
hsa‐miR‐199b‐3p	7.58	8.63	2.07	MICT
hsa‐miR‐432‐5p	2.36	3.43	2.1	MICT
hsa‐miR‐7110‐5p	3.13	4.21	2.11	HIIT
hsa‐miR‐10a‐5p	3.71	4.8	2.12	HIIT/MICT
hsa‐miR‐497‐5p	3.04	4.15	2.15	HIIT/MICT
hsa‐miR‐3613‐5p	3.3	4.41	2.16	HIIT/MICT
hsa‐miR‐487b‐3p	2.18	3.29	2.16	MICT
hsa‐miR‐4284	5.67	6.81	2.21	MICT
hsa‐miR‐499a‐5p	3.74	4.89	2.21	MICT
hsa‐miR‐664b‐3p	1.16	2.31	2.22	HIIT
hsa‐miR‐505‐5p	1.22	2.39	2.24	HIIT
hsa‐miR‐139‐3p	1.97	3.14	2.25	HIIT/MICT
hsa‐miR‐3180‐3p	2.8	3.99	2.28	HIIT/MICT
hsa‐miR‐433‐3p	1.14	2.45	2.48	MICT
hsa‐miR‐134‐5p	1.43	2.75	2.5	HIIT
hsa‐miR‐34a‐5p	0.84	2.17	2.51	MICT
hsa‐miR‐146b‐5p	1.11	2.56	2.73	HIIT
hsa‐miR‐424‐3p	1.81	3.26	2.73	MICT
hsa‐miR‐615‐3p	1.56	3.08	2.87	MICT
hsa‐miR‐708‐5p	0.87	2.45	3	HIIT/MICT
hsa‐miR‐503‐5p	2.39	4.07	3.21	HIIT/MICT
hsa‐miR‐4720‐5p	0.81	2.51	3.25	MICT
hsa‐miR‐382‐5p	1.74	3.49	3.36	MICT
hsa‐miR‐26b‐5p	1.67	3.5	3.56	HIIT/MICT
hsa‐miR‐21‐5p	2.19	4.67	5.58	HIIT/MICT
hsa‐miR‐3613‐3p	1.34	4.29	7.76	HIIT/MICT

Note

^a^ This miR was selected by the TAC Expression Console algorithms despite the fact that plain numerical values for both the three MICT and the three HIIT subjects narrowly did not meet the selection criterion of a minimum fold change of −2/2.

To study the involvement of differentially expressed miRs in cellular functions, we carried out KEGG pathway analysis (Vlachos et al., 2015). The results suggest that one major level at which adaptation to our protocol of endurance exercise occurs might be fatty acid turnover, but also other pathways, such as ECM‐receptor interaction (Table 5).

**TABLE 5:** Pathway analysis

KEGG pathway	*p* value	Genes	miRNAs
1. Fatty acid biosynthesis (hsa 00061)	<1e^−325^	4	5
2. ECM‐receptor interaction(hsa04512)	<1e^−325^	21	6
3. Prion diseases (hsa05020)	2.220446e^−16^	1	1
4. Fatty acid metabolism (hsa(01212))	2.220446e^−16^	27	7
5. Proteoglycans in cancer (hsa05205)	2.220877e^−09^	107	5
6. Lysine degradation (hsa00310)	4.632769e^−07^	22	9
7. Hippo signaling pathway (hsa04390)	3.145942e^−05^	67	6
8. Adherens junction (hsa04520)	0.0003716131	45	6
9. p53 signaling pathway (hsa04115)	0.03928435	53	6

Note

Functional analysis of differentially expressed miRs was carried out using KEGG pathway analysis.

**FIGURE 3:**
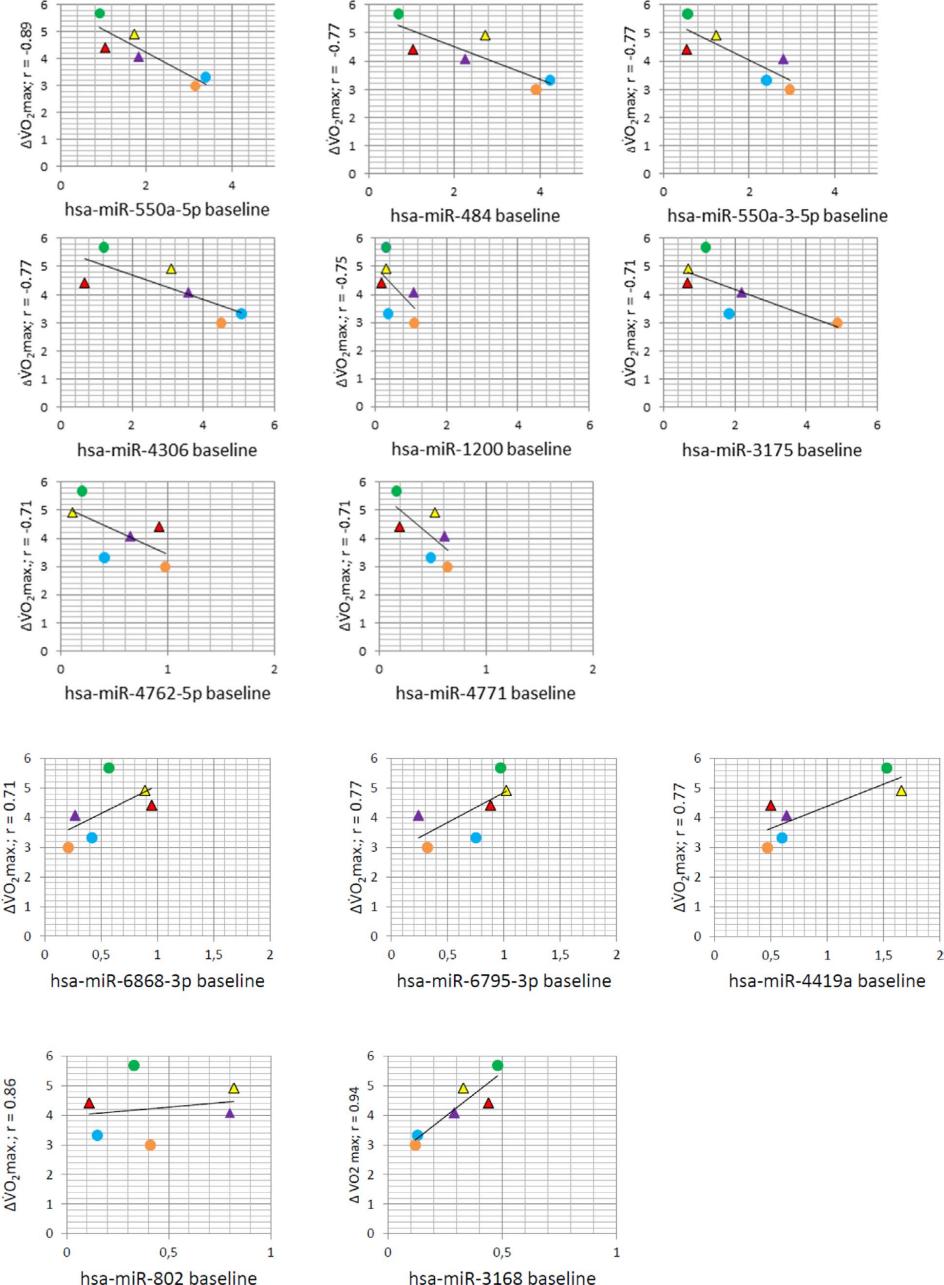
Correlation of baseline miR concentrations in skeletal muscle samples of all six subjects and ΔVO_2_max. miRs were screened for a potential correlation of baseline expression levels and gains in VO_2_max (ml kg^−1^ min^−1^) with training. Data for miRs with correlation coefficients of <−0.7 or >0.7 are shown. Circles mark subjects that performed MICT, triangles subjects that performed HIIT training

Next, as a second arm of the study, we aimed at making a first attempt at identifying potential correlations between baseline levels (log2‐transformed) of specific miRs and gains in VO_2_max, since such miRs might serve as biomarkers for the prospective design of individualized training regimens. Here, we identified 13 miRs, ‐550a‐5p, ‐484, ‐550a‐3‐5p, ‐4306, ‐1200, ‐3175, ‐4762‐5p, ‐4771, ‐6868‐3p, ‐4419a, ‐6795‐5p, ‐802, and ‐3168, which showed a major correlation (*r* < −0.7 or >0.7) with ΔVO_2_max (Figure 3). These can now be tested and evaluated in larger cohorts in the future.

## 4 DISCUSSION

miRs might be important biomarkers for the design of individualized exercise regimens. However, the establishment of suitable marker patterns is difficult, specifically since a plethora of different miR species have been identified over the last few years, with probably more to be discovered in the future. The development of miR arrays has allowed fast, comprehensive, and unbiased screening of a specific sample for more or less all miRs known at that time. Expression data can then be correlated with individual physiological outcomes, such as, as in our study, changes in VO_2_max as an indicator of training adaptation. The goal of the explorative study described here was the identification of miR patterns that might be good candidates for individualized training biomarkers, and for which further testing in larger, confirmatory studies in the future might be worthwhile.

However, as a first step, specifically, to view our results in the context of literature data, we did a biased approach, which means that we analyzed the expression of specific miRs which had previously been described in the literature as being differentially expressed in response to exercise. Specifically, we studied expression of miRs ‐1, ‐21, ‐133a, and 133b. Consistent with previous results obtained in other tissues, namely heart and blood cells, and in the circulation (for review, see Sapp et al., 2017; Silva et al., 2017, 2020; Widmann et al., 2019), miR‐21 was strongly upregulated in response to training in skeletal muscle tissue derived from subjects of both training groups. By contrast, there was no regulation of miR‐1, ‐133a, and ‐133b expression. These differences might be due to differences in the experimental and analytical design of our trial and other studies. One example is the study by Cui et al. (2016), where subjects underwent two single, acute bouts of running exercise (HIIT and MICT), with miRs being assessed in plasma: Here, induction of both miRs‐1 and ‐133a/b could be detected. Similarly, for the study described by Ramos et al. (2018), participants did four single sessions of persistent graded‐intensity running protocols, with sessions being spaced by one week and myomiRs being assessed in the circulation: The authors describe the induction of miRs‐1 and ‐133a, but not of miR‐21. The focus of the study by Fyfe et al. (2016), by contrast, was concurrent training: Whereas in line with our study, the authors analyzed skeletal muscle tissue, their exercise protocol was largely different, consisting of three single, consecutive bouts of resistance exercise, two of them combined with HIIT or MICT cycling, respectively, resulting in reduced levels of miR‐133a. Finally, most studies analyzed all‐male cohorts, in contrast to our all‐female group, and both subjects’ ages and fitness levels differ significantly between individual studies. Re‐analysis of the array data using qPCR showed similar results for miR‐1 and miR‐21, in case of the latter also in a larger cohort of subjects. By contrast, for miRs ‐133a and ‐133b, we observed consistent downregulation, a finding that has previously been described by others (Gargalionis & Basdra, 2013; Luo et al., 2013; McCarthy, 2011). Overall, these data suggest that miR array analysis of a small subset of samples might be an efficient pre‐screening tool to characterize exercise‐induced miR patterns in skeletal muscle tissue. The results can then be verified by qPCR analysis in larger cohorts.

Next, we analyzed our data using an unbiased screening strategy, selecting miRs that were, on average, up‐ or downregulated at least twofold by training. Using this approach, we could identify several miRs previously implicated in skeletal muscle plasticity, metabolism and exercise adaptation, namely the already‐mentioned miR‐21‐5p, but also miRs ‐26b‐5p (Margolis et al., 2017), ‐382‐5p (Dahlmans et al., 2019), ‐503‐5p (Shen et al., 2013; Wang et al., 2019), ‐708‐5p (Baghdadi et al., 2018), ‐615‐3p (Siengdee et al., 2015), ‐146b‐5p (Khanna et al., 2014), ‐424‐3p (Gonzalo‐Calvo et al., 2015), ‐34a‐5p (Gonzalo‐Calvo et al., 2015), ‐505‐5p (Mach et al., 2016), ‐499a‐5p (Wang et al., 2017; Xu et al., 2018), ‐487b‐3p (Katase et al., 2015; Wang et al., 2018; Zhang et al., 2018), ‐497‐5p (Sato et al., 2014; Wei et al., 2016), ‐432‐5p (Dmitriev et al., 2013), ‐379‐5p (Gao et al., 2015), ‐199a‐3p (Zhang et al., 2020), and ‐199b‐3p (Zhu et al., 2019). In addition, we found that several of the differentially expressed miRs were related to vascular biology, specifically miRs ‐503‐5p (Caporali et al., 2011), ‐487b‐3p (Nossent et al., 2013; Welten et al., 2014), ‐26b‐5p (Martello et al., 2018), and ‐199b‐3p (Chen et al., 2015). Furthermore, beyond these, we also identified a variety of other differentially expressed miR species which might be candidates for future testing as potential markers of skeletal muscle exercise adaptation and plasticity in general. In this context, specifically, miR‐3613‐3p, which we found to be strongly induced in response to exercise, might be interesting: This variant is produced from an intron that belongs to a long non‐codingRNA (DLEU (deleted in lymphocytic leukemia) 2) (https://www.genecards.org/cgi-bin/carddisp.pl?gene=DLEU2). The DLEU2 lncRNA has been recently related to sarcopenia, acting as a sponge of another miRNA (hsa‐miR‐181a) (Wang et al., 2020). Comparable to the abovementioned results, for some of these data, specifically results obtained for miRs‐487‐3p and ‐503‐5p, subsequent qPCR analysis showed a similar overall trend, whereas other analyses yielded inconsistent data, namely those for miRs ‐379‐5p and ‐497‐5p, suggesting again that microarray analysis might be a good screening tool with regard to miR changes in human skeletal muscle in response to exercise, generating hypotheses, which should then be tested, verified and refined using alternative methods. Induction of miR‐503‐5p with exercise could already be confirmed in this study. This species is a member of the miR‐15/‐107 miR family and has been suggested to play a role in skeletal, cardiac, and smooth muscle cell differentiation and atrophy, as well as in endothelial cells and angiogenesis (Shen et al., 2013; for review, see Wang et al., 2019). Thus, due to its potential involvement in several aspects of exercise adaptation, miR‐503‐5p, in particular, might be an interesting candidate for future functional and mechanistic studies.

Furthermore, despite the exploratory character of our analysis and the small number of subjects, our array data and also qPCR data generated for miRs ‐21‐5p and ‐503‐5p indicate that some miRs might be rather responsive to MICT or HIIT, respectively, whereas others appeared to react to both types of training. These findings give the first hint that different training regimens might indeed induce differential miR profiles, a finding that should be further analyzed in larger and possibly confirmatory studies in the future, especially since some studies suggest that training intensity (and maybe not the intermittent nature of interval protocols) might be the most important factor for most physiological adaptation reactions, also outclassing training volume or total workload (MacInnis & Gibala, 2017; Tjønna et al., 2013). In addition, to study the obvious and not‐yet‐addressed question of whether adaptation to MICT and HIIT might be different at the molecular level, despite evoking similar increases in VO_2_max, it would be very interesting to carry out further KEGG pathway analyses with a larger set of data. Our initial analysis, which, due to the small number of subjects, did not discriminate between MICT and HIIT, had suggested that adaptations might specifically have taken place at the level of fatty acid turnover, a metabolic pathway that is not unlikely to be differentially regulated by HIIT versus MICT: It is very likely that different patterns of metabolic demand might induce differential miR patterns, which might then lead to unique and regimen‐typical changes in gene expression patterns, for example with regard to genes encoding metabolic regulators, despite the fact that both regimens bring about an increase in aerobic fitness.

Unfortunately, despite the fact that there was a break of at least 48h between the last training session and FU1 analysis, we cannot completely rule out the possibility of acute effects of this session on FU1 miR patterns. However, since other studies on acute training effects indicate that most differentially expressed miRs return to baseline levels within this time frame (Gargalionis & Basdra, 2013; Luo et al., 2013; McCarthy, 2011), a major contribution of acute effects is unlikely.

Overall, our data demonstrate that despite the existence of general regulatory motifs, miR patterns in skeletal muscle and response to exercise show a high degree of inter‐individual variability, thus suggesting that they might be suitable markers to predict individual training responses in a specific setting, i.e., to serve as a prognostic marker in the planning of individualized training regimens in the future. To this end, specifically correlations of baseline miR expression parameters and training outcomes, such as gains in VO_2_max, will be interesting. As a first attempt to establish such patterns, baseline expression data of all miRs were correlated with gains in VO_2_max of each individual subject. Using this strategy, we found correlations for miRs ‐550a‐5p, ‐484, ‐550a‐3‐5p, ‐4306, ‐1200, ‐3175, ‐4762‐5p (*r* < −0.7), ‐4771, ‐6868‐3p, ‐4419a, ‐6795‐5p, ‐802, and ‐3168 (*r* > 0.7). Most of them have not been implicated in skeletal muscle plasticity and/or exercise adaptation so far. However, there are some data on miR‐4419a upregulation in saliva in response to long‐distance running (Hicks et al., 2018). Furthermore, in fibroblasts, miR‐550a‐5p has been shown to be induced by metabolic stress (Kálmán et al., 2014), a feature that might also be important in skeletal muscle exercise adaptation. Finally, miR‐550a‐3‐5p appears to be a potent regulator of Yes‐associated protein (YAP; Choe et al., 2018), a central component of the Hippo pathway which is a major player in skeletal muscle hypertrophy and exercise adaptation (for review, see Gabriel et al., 2016). The results of our initial, exemplary screen will now have to be extended, evaluated, refined, and tested in prospective studies with larger cohorts of subjects. In addition, depending on the specific goal of the exercise program in question, it might be worthwhile to correlate miR patterns with physiological changes other than VO_2_max: Parr et al. (2016) were able to identify differential patterns of (circulating) miRs in high and low responders to a diet‐ and exercise‐based lifestyle intervention program. Moreover, a recent publication derived differential miR patterns in response to sprint interval training from leukocytes and correlated them with leukocyte telomere length, which is again correlated with individual aerobic fitness (Kumar Dev et al., 2021).

## 5 LIMITATIONS OF OUR STUDY

Our project was designed as an explorative screening study and, due to the low number of subjects, does not allow extensive statistical evaluation beyond simple correlation analysis. Its major aim was to develop strategies to generate hypotheses for further, confirmatory analyses. The first hypotheses we generated, were based on the miRNA array analysis. Since the related costs did not allow broad array screening of all participants, we gathered some preliminary ideas via array analysis and then validated them by qPCR. Now, these have to be phrased and tested with larger subject numbers and also different cohorts divergent for age, sex, basal activity level, health status, hormonal situation, especially in pre‐menopausal females as analyzed in this study, and, particularly, training regimen, and statistically analyzed, including corrections for multiple testing. In addition, miR patterns will have to be confirmed using alternative methods, such as semi‐quantitative RT‐PCR (qPCR), at a broader range. As a complementary approach, it might also be promising to analyze the acute response to a single bout of exercise and correlate these data with the physiological effect achieved by training. Furthermore, in routine clinical practice and also in high‐performance, competitive sport, it will usually not be feasible to obtain skeletal muscle data. To this end, similar studies analyzing patterns of circulating miRs, have to be carried out (for review, see Polakovičová et al., 2016; Sapp et al., 2017). However, since circulating miRs originate from a broad variety of tissues and organs, their concentrations in the circulation are potentially less stable and more prone to external disturbances when compared to a single tissue such as skeletal muscle, suggesting that it will be more challenging to establish reliable sets of markers. Finally, our current data set does not allow mechanistic approaches to the roles of miRs in skeletal muscle training adaptation. For this purpose, functional tests, such as murine knockout studies, will have to be performed. In addition, more extensive regulatory network analysis, using appropriate bioinformatics tools, might provide insight into functional interrelationships between individual miRs and/or their target genes in skeletal muscle training adaptation.

## CONFLICT OF INTEREST

The authors declare no conflict of interest.

## AUTHOR CONTRIBUTIONS

Conceptualization, B.M. and A.M.N.; Methodology, M.W., F.M.M., C.B., G.E., P.S., A.F., A.S., and B.M.; Software, M.W. and F.M.M.; Formal Analysis, M.W., F.M.M., and B.M.; Investigation, M.W., F.M.M., C.B., G.E., P.S., A.F., A.S., A.M.N., and B.M.; Resources, B.M. and A.M.N.; Data Curation, M.W., F.M.M. and B.M.; Writing – Original Draft Preparation, B.M. and M.W.; Writing—Review & Editing, M.W., F.M.M., C.B., G.E., P.S. A.M.N., and B.M.; Visualization, M.W. and B.M.; Supervision, A.M.N. and B.M.; Project Administration, A.M.N. and B.M.; Funding Acquisition, A.M.N. and B.M.
